# Characterization of Probiotic Properties of *Lacticaseibacillus paracasei* L2 Isolated from a Traditional Fermented Food “Lben”

**DOI:** 10.3390/life13010021

**Published:** 2022-12-21

**Authors:** Amina Cheikh M’hamed, Karima Ncib, Abderrahmen Merghni, Mariem Migaou, Houda Lazreg, Mejdi Snoussi, Emira Noumi, Mohamed Ben Mansour, Raoui Mounir Maaroufi

**Affiliations:** 1 Laboratory of Genetics, Biodiversity and Valorization of Bio-Resources (LR11ES41), Higher Institute of Biotechnology of Monastir, University of Monastir, Avenue Tahar Haddad, BP74, Monastir 5000, Tunisia; 2 Laboratory of Analysis, Treatment and Valorization of Pollutants of the Environment and Products, Faculty of Pharmacy, University of Monastir, Avenue Tahar Haddad, BP74, Monastir 5000, Tunisia; 3 Laboratory of Antimicrobial Resistance LR99ES09, Faculty of Medicine of Tunis, University of Tunis El Manar, Tunis 1002, Tunisia; 4 Department of Biology, College of Science, Hail University, P.O. Box 2440, Hail 2440, Saudi Arabia

**Keywords:** *Lacticaseibacillus paracasei*, probiotics properties, Lben, antimicrobial, antioxidant

## Abstract

Lben is a dairy fermented food that is largely consumed in Tunisia for its numerous health benefits that are related to the existence of probiotics. Lactic Acid Bacteria (LAB) are well known for their beneficial probiotic properties for humans, especially when administered in adequate amounts. The aim of this study was to isolate and investigate the probiotics properties of *Lacticaseibacillus paracasei* L2 from Lben. The isolated strain was identified by 16S r-RNA gene sequences and MALDI- TOF MS. To evaluate the probiotic potential of the isolated bacterium, in vitro tests were performed, including adhesion ability to HCT-116 cells, survival in acid and bile salt conditions, lysozyme resistance, biofilm formation, hemolytic activity, antioxidant activity, and antimicrobial activity. Our results revealed that the selected *Lacticaseibacillus paracasei* L2 strain expressed a high adherence to HCT-116 cells (45.03%), survived under acidic conditions (pH3), and showed a resistance to bile salts. The strain was considered as safe (α-hemolysis). *L. paracasei* L2 showed a high biofilm-formation ability (OD 570 > 1.7) after 24 h of incubation. It also demonstrated an important antioxidant activity in the range of 85.31% for the intact cells. However, an antimicrobial activity against pathogens, namely *Staphylococcus aureus*, was detected with an IZ that was above 19 mm. In conjunction with the results obtained and the technological properties of *Lacticaseibacillus paracasei* L2 (proteolytic property, autolytic activity, acidifying activity, and EPS production), this strain may be used as a probiotic for manufacturing fermented foods.

## 1. Introduction

According to the World Health Organization [1], probiotics are defined as living microorganisms within hosts that exert health benefits when ingested in sufficient amounts. They play an important role in host health because they have nutritional, immunologic, and physiological functions. Probiotics also play a role in regulating the mucosal and systemic immunity. Nowadays, probiotics research and applications are increasing on a global scale [2].

Probiotics have shown many health benefits. In order to exert their positive outcomes they must remain viable in the acidic conditions of the gastro intestinal tract (GIT) [3]. These microorganisms are known to be useful not only for their ability to adjust the intestinal balance of the host but also for their protective effects against gastrointestinal pathogens, using various anti-microbial mechanic techniques such as organic acid production. The ingestion of probiotics alleviates the pathological condition of oxidative stress-related model diseases. Oxidative stress is associated with many diseases, such as cancer, diabetes, heart disease and neurological disorders [4].

Furthermore, probiotics have demonstrated anti-inflammatory properties against bowel diseases as well as anticancer effects in “in vivo” systems [5]. Additionally, other studies have shown that probiotics can have diverse functional effects, such as anti-diabetic, anti-allergy and anti-inflammatory effects that can improve host health [6]. Moreover, they have manifested several health benefits, such as enhancing immune function, lowering serum cholesterol, and treating many intestinal disorders, such as inflammatory bowel diseases and allergic responses [7].

Recently, lactic acid bacteria (LAB) have been “generally recognized as safe GAS” by the FDA (US food and drug administration) and have received the “Qualified Presumption of Safety” status by the ESFA (European food authority) [8]. They have been widely investigated for their probiotic properties. LABs have also been investigated in relation to their essential role in fermented foods as well as their ability to produce various antimicrobial compounds that promote probiotic properties. These properties include antitumor activity, reduction of serum cholesterol, alleviation of lactose intolerance, stimulation of the immune system, stabilization of gut microflora and production of exopolysaccharides [9,10,11]. LAB are a heterogeneous group characterized as gram positive, usually non-motile, non-sporulating short-rod, catalase-negative, anaerobic, and aero-tolerant that produce lactic acid as a major result of fermentative metabolism [12,13]. They grow strongly under anaerobic conditions. They may also grow under microaerophilic as well as aerobic conditions. Moreover, at slightly lower acidic conditions (pH 5.5–6.0), LAB strains exhibit optimum growth [10].

Lactic acid bacteria are widely distributed in nature. They are found mainly in the digestive system, plants, and fermented foods, including dairy products, yogurt, meats, and alcoholic beverages. [10]. Several probiotics have been isolated from dairy products as well as from the human digestive tract [8]. The most studied strains of microorganisms that meet the criteria of a probiotic are of the genre of a *Lactobacillus* and *Bifidobacterium*. However, other strains that include the *Streptococcus*, the *Enterococcus*, and yeasts also have a probiotic action [14].

Kakerlar et al. (2019) noted that the *Lactobacillus* species are the most important probiotic bacteria, which are linked to the natural microflora. Due to their tolerance to bile and acid, they are compatible with the human gastrointestinal system [15]. Furthermore, they play a crucial part in preserving the ecological balance among the different species of microorganisms. Additionally, because of their role in the production of fermented foods, their use as probiotics [16], their anti-inflammatory properties, their antibacterial effects [17], and their antifungal properties [13], lactobacillus are significant bacteria that are involved in food microbiology mechanisms and human nutrition. 

New probiotic bacteria are isolated and identified from various natural sources, including the gastrointestinal system, plants, fermented meals, and dairy products [10,18]. In this context, the present study was conducted in order to isolate, identify, and characterize new lactic acid bacteria from a homemade dairy fermented product called “Lben”.

## 2. Materials and Methods

### 2.1. Isolation of Strains

Lben was used as a sample for the isolation of the strains. The serially diluted samples in PBS (phosphate-buffered saline) were added to MRS (de Man, Rogosa and Sharpe) medium (Accumix, Spain). A total of four strains were isolated on MRS agar at 37 °C for 24 h to 48 h. Single pure rod-shaped colonies were subjected to standard morphological biochemical tests. Catalase-negative and gram-positive strains were selected for identification.

### 2.2. Identification of Lactic Acid Bacteria Strain

The MALDI-TOF MS (Matrix Assisted Laser Desorption Ionization-Time of Flight mass spectrometry) and the16S r-RNA sequencing analysis were used to identify the strain.

MALDI-TOF MS identification

After incubation, for 24 h to 48 h at 37 °C, a sample preparation was made according to Noumi et al. [19].

Sequencing and analyzing the 16Sr-DNA gene

A molecular confirmation of the identification of the selected LAB strain was carried out. The entire genomic DNA of the strain was extracted, using the Genomic DNA purification kit “All in one INC Bio basic”, according to the manufacturer’s instructions. The 16S r-DNA amplified with the universal bacterial primers 27f/1492r. The fragments were amplified in a MultiGene Thermocycleur (Labnet International) under the following conditions: 35 cycles of 94 °C for 5 min, 55 °C for 30 s, 72 °C for 90 s and finally 72 °C for 10 min. The amplified fragment was screened on agarose gel and sequenced by the Tunisian RAN Biolinks Company, Tunis, Tunisia. Results from the bacterial identification of the LAB sequencing were analyzed by searching the database of The National Center for Biotechnology Information Nucleotide, using the BLAST website (http://blast.ncbi.nlm.nih.gov) accessed on 15 December 2021. A sequence alignment was performed via BioEdit, and a phylogenetic tree was generated via MEGA v6.06.

### 2.3. Survival under GIT (Gastro Intestinal Transit) Conditions

Acid tolerance

The ability of the strain to survive under acid conditions at pH 2, pH 2.5, pH 3, pH 4 and pH 6 was evaluated by the method of Oh et al. [20]. Acid tolerance was evaluated by a plate count on MRS agar. The viable cells were counted at 0 h and after 2 h, 3 h of incubation at 37 °C. The number of viable colonies is expressed as a survival rate according to the following formula:

Survival rate % = [cell number (log CFU/mL) survived in MRS Containing HCl/cell number (log CFU/mL) of initial inoculated Cell] × 100.

Bile salt tolerance

The evaluation of bile salt tolerance of the strains that survived under acid tolerance for 3 h was done following the method described by Oh et al. [20], using MRS broth containing 0.3 0.5 et 1% (*w/v*) of ox gall (Sigma-Aldrich, St Louis, MO, USA). The bile salt tolerance was evaluated by measuring the survival cell count, using MRS agar after 24 h and 48 h of incubation at 37 °C. The results are expressed according to the following equation:

The survival rate % = [cell number (log CFU/mL) survived in MRS Containing 0.3% ox gall/cell number (log CFU/mL) of the initial inoculated Cell] × 100

Lysozyme resistance

Lysozyme resistance was assessed following an adapted version of Dias et al. method [21] with some modifications. The bacteria were grown on MRS that was supplemented with commercial lysozyme (Thermo Scientific™, Oxford, UK) at different concentrations (100, 200, 300 and 500 mg/mL (*w/v*)). Bacteria count was measured at 0 h, 3 h, and 6 h and after 24 h of incubation at 24 h. 

The survival rate % is calculated according to the following equation:

The survival rate % = [cell number (log CFU/mL) survived in MRS Containing 100 mg/ml lysozyme/cell number (log CFU/mL) of initial inoculated Cell] × 100.

NaCl resistance

Salt tolerance was determined following Seixas et al. method [22]. Briefly, 100 mL of modified MRS broth containing 2%, 4%and 6.5% NaCl were inoculated (1% *v/v*) with overnight cultures in MRS broth and incubated for 24 h at 32 °C. Then the OD 595 (Optical Density) was measured. The cultures in MRS broth were used as control. 

The results were expressed as the ratio OD595 in modified MRS/OD595 in MRS.

### 2.4. Safety‘s Tests

Antibiotic resistance test

The resistance of the LAB strain to antibiotics was performed according to the disc diffusion method. The resistance was screened against streptomycin (10 mg/mL), erythromycin (15 mg/mL), tetracycline (30 mg/mL), gentamicin (10 mg/mL), kanamycin (30 mg/mL), penicillin (10 mg/mL), ciprofloxacin (5 mg/mL), and nalidixic acid (30 mg/mL). The LAB strains (10^6^ CFU/mL) were spread onto MRS agar, and antibiotic discs were set on the MRS agar surface. The diameters (mm) of the inhibition zone were measured after 24 h of incubation at 37 °C.

Hemolysis

The LAB isolate grown for 24 h in MRS broth was transferred onto Tryptic Soy Agar (NutriSelect^®^ Plus) containing 5% (*w/v*) sheep blood (Jan H et al., 2019) and incubated for 24 h at 37 °C. Hemolytic activity was assessed by observing clear hydrolysis zones around colonies (β-hemolysis), partial hydrolysis and green-hued zones around colonies (α-hemolysis), or no zones around colonies (γ-hemolysis). γ hemolysis was considered negative.

### 2.5. Antimicrobial Activity

The spot-on-lawn method was performed to evaluate the antimicrobial activity of the isolated strain against nine pathogenic strains: *Listeria monocytogenes* ATCC19115, *Micrococcus luteus* NCIMB 8166, *Enterococcus faecalis* ATCC 29212, *Bacillus cereus* ATCC 11778, *Staphylococcus aureus* ATCC 25923, *Pseudomonas aeruginosa* ATCC 27853, *Escherichia coli* ATCC 35218, and *Salmonella Typhi* ATCC 1408. The bacterial suspension of each pathogen adjusted to 10^7^ CFU/mL was dipped into a cotton swab and streaked across the surface of Mueller–Hinton (MH) agar medium. To evaluate antimicrobial activity, LAB strain (approximately 10^9^ CFU/mL) was spotted onto plates and incubated at 37 °C for 24 h. Clear colonies with translucent halo in the upper layer were considered positive for the antimicrobial activity. The clear zones were measured and represented as diameters (mm) inhibition zone (IZ). The clear colonies presenting a translucent halo in the upper layer were considered positive for the antimicrobial activity. The clear zones were measured and represented as diameters (mm) inhibition zone (IZ).

### 2.6. DPPH Free Radical Scavenging Ability

The selected strain was cultured at 37 °C for 24 h, then centrifuged (14,240× *g*/5 min/4 °C), and finally washed twice with PBS. The bacterial cells were resuspended in PBS to 10^7^ CFU/mL.

The DPPH (2,2-Diphényl 1-picrylhydrazy) radical scavenging assay was measured by an adapted version of Valan arasu et al. method [13] with some modifications. First, 1 ml of the re-suspended LAB strain (10^7^ CFU/mL) was added to 3 mL of the DPPH solution. The mixture was shaken and reacted for 30 min in the dark. The experiment was conducted in triplicate.

The absorbance was measured at 517 nm. The scavenging rate of DPPH radicals was described as follows:

DPPH radical scavenging activity % = (1 − A sample/A control) × 100

With:

A sample: absorbance of the sample at 517 nm.

A control: absorbance of the control at 517 nm.

### 2.7. Investigating Enzymatic Activity

The strain was also analyzed for enzyme production using the API ZYM kit (BioMerieux, Marcy-l’Etoile, France). The LAB strain was incubated in MRS broth at 37 °C for 24 h and then centrifuged (14,240× *g*, 5 min, 4 °C). The cell pellet was re-suspended in PBS, and 65 µL (106 CFU/mL) was inoculated into each cupule.

After incubation at 37 °C for 4 h, ZYM A and ZYM B reagents were sequentially dropped into each cupule. Enzyme production was evaluated. The sample’s color changed, indicating that the substrate had been hydrolyzed. Enzymatic activity was evaluated according to the color reaction chart.

The enzymatic activity was graded between 0 and 5 (0: no activity; 1: liberation of 5 nmol; 2: 10 nmol; 3: 20 nmol; and 4: 30 nmol) [23].

### 2.8. Auto-Aggregation

The auto-aggregation abilities of the selected isolate were analyzed following Lee et al. [5].

After the centrifugation, the LAB cells were washed twice with PBS and re-suspended in the same buffer. The OD were adjusted (OD600 nm = 0.25 ± 0.05). Each bacteria suspension (4 mL) was incubated at 37 °C for 24 h. The absorbance was read at 600 nm at 2 h intervals of incubation. Auto-aggregation was expressed as follows:

The percentage was expressed as auto-aggregation %:

Auto-aggregation % = 1 − (At/A0)

where:

A0: represents the absorbance at 0 h.

At: represents the absorbance at 2, 4, 6, 12, and 24 h.

### 2.9. Cell Surface Hydrophobicity

The bacterial adhesion to the solvents was carried out according to Jan H et al. [17]. To characterize the cell surface hydrophobicity of the strain, three solvents were used: Xylene was used as an apolar solvent, chloroform as an electron acceptor (monopolar and Lewis-acid solvent), and ethyl acetate as an electron donor (monopolar and Lewis-base solvent). The LAB was incubated in MRS broth at 37 °C for 24 h. These cultures were centrifuged at 14,2409× *g* for 5 min; then the pellet was washed twice and re-suspended in PBS. The OD of the re-suspended cells was adjusted to 0.5 (OD Initial). 3 mL of the re-suspended cells was mixed with 1 mL of each solvent and pre-incubated for 10 min at 37 °C. The mixture was mixed for 1 min and incubated at 37 °C for 20 min. After incubation, the mixture was separated into two phases. Absorbance of the aqueous phase was measured at 600 nm (OD time).

The cell surface hydrophobicity was expressed as follows:

Cell surface hydrophobicity (%) = (1 − ODTime/OD Initial) × 100

where:

OD Time: absorbance was measured at 600 nm.

OD Initial: absorbance initial measured at 600 nm.

### 2.10. Production of Exopolysaccharides

The LAB isolate was plated on an MRS agar with sucrose as the only carbon source. The plates were incubated at 37 °C for three days. Triplicate plates were performed. The colonies were scored for their mucoid property.

### 2.11. Biofilm Quantification

Phenotypic characterization of bacteria-producing slime

Identification of slime-producing strains was performed by culturing isolates on Congo red agar (CRA). Isolated strains were incubated at 37 °C for 24 h under aerobic conditions. Colonies with black color and rough surfaces were identified as slime producers and those with red and smooth surfaces as non-slime producers.

Biofilm production was assessed by crystal violet staining assay on 96-well tissue culture plates, as described previously by Vandecandelaere et al. [24]. Adherent bacteria were fixed with 95% ethanol and stained with 100 μL of 1% crystal violet for 5 min at room temperature. The microplates were air-dried, and the optical density was measured of each well at 570 nm (OD570), using Thermo Scientific Multiskan FC Microplate.

Biofilm formation was interpreted as highly positive (OD570 ≥ 1), low grade positive (0.1 ≤ OD570 < 1), or negative (OD570 < 0.1).

### 2.12. Adhesion Ability to the HCT-116 Cells

The bacterial adhesion ability was carried out using the method of Jeon et al. [6]. The human colon carcinoma cells (HCT-116) were used in this test. HCT-116 cells were seeded into 24-well polystyrene plates at a concentration of 1 × 10^5^/well and allowed to change for three days at 37 °C in a 5% CO_2_ incubator. The medium was changed daily. An overnight culture was prepared and centrifuged and then resuspended in PBS to an appropriate dilution. The bacterial cells were added to each well. The plate was incubated at 37 °C for 2 h and then washed three times with PBS. Later, 1 mL of 1% (*v/v*) Triton X-100 (Sigma-Aldrich) solution was added to detach the bacteria during incubation for 10 min. The detached strains were serially diluted and spread over the MRS agar plates. The plates were incubated at 37 °C for two days. The percentage of bacterial adhesion was calculated according to the following equation:

Adhesion% = (adhered bacteria/total added bacteria) × 100

### 2.13. Technological Characterization

Acidifying activity

This test was performed following the method that was described by Seixas et al. [22]. An overnight culture was prepared in the MRS broth at 37 °C. Tubes containing 10 mL of sterile skimmed milk were inoculated (1% (*v/v*)) with revitalized strains and incubated at 37 °C. pH was measured after 6 and 24 h of incubation and values were expressed as ΔpH.

Autolytic activity

The Seixas et al. [22] method was used to measure the autolytic activity. The lysis of strains was tracked during 4 h incubation at 30 °C by recording the decrease in OD650 using a spectrophotometer (spectrophotometer T70 UV/VIS). The percentage of lysis was determined as:

% lysis = 100 − (A1/A2 × 100)

where:

A1: is the lowest value of the OD650 measured during incubation.

A2: the highest value of the OD650 measured during incubation.

### 2.14. Statistical Analysis

The data for each treatment were assigned in triplicate and expressed as a mean ± standard deviation. Data were tested for normality to verify the model assumptions using analysis of variance (ANOVA) and treatments means were separated by Duncan’s multiple range test (*p* < 0.05) using SAS 2002 software version 9.

## 3. Results

### 3.1. Isolation and Identification of Lactobacilli from Lben

**Figure 1:**
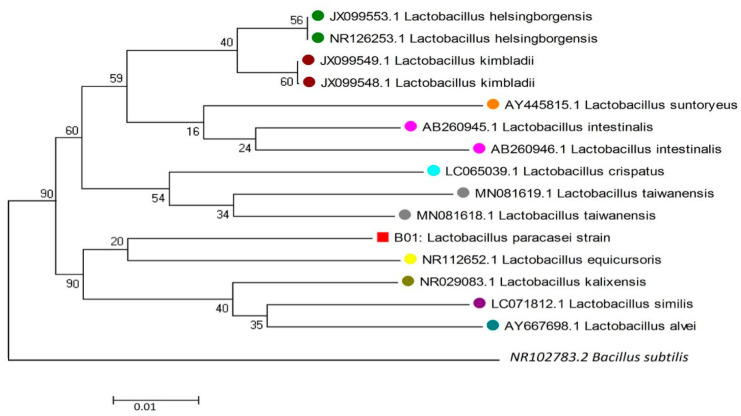
The phylogenetic tree was built on the basis of 16S r-DNA sequences. The scale bar 0.01 indicates the nucleotide substitution rate at each site. Bootstrap probabilities were presented as the percentage values and determined using 1000 replicates. Before the strain name, the existing numbers represent the accession numbers of selected sequences. The colored circles indicate the strains from NCBI and the square indicates the selected strain. The Empty circle is the out groups used for tree construction.

A total of four strains were isolated from the lben. Only two showed a resemblance to lactic acid bacteria on an MRS agar Medium. During the classification of the differential staining by Gram’s method, the isolates were identified as gram-positive and rod-shaped cultures. All the isolates were categorized as catalase negative. All the isolated strains were identified by MALDI-TOF. Only one *bacterium* (L2) was confirmed as belonging to the *Lactobacillus* genus. L2 was selected for the molecular identification by the 16 r-DNA sequencing. A phylogenetic tree was generated based on its 16 r-DNA sequence, using a neighbor-joining analysis (Figure 1).

Results showed that the isolated LAB strain belonged to *Lacticaseibacillus paracasei*, with 99% similarity to *L. paracasei*.

### 3.2. Survival under GIT Conditions

Acid tolerance

**Figure 2:**
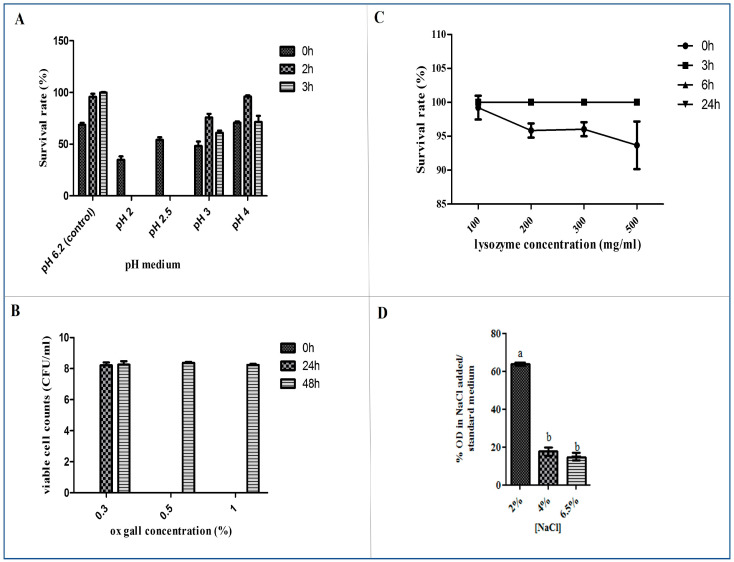
(**A**) survival rate of *L. paracasei* L2 isolated from Lben after incubation for 3 h in MRS broth at pH 2, 2.5, 3 and 4; (**B**) the viable counts of *L. paracasei* in MRS broth supplemented with 0.3%, 0.5% and 1% bile (ox gall); (**C**) survival rate of *L. paracasei* L2 after incubation for 24 h in MRS broth supplemented with various concentrations of lysozyme (100 mg/L, 200 mg/L, 300 mg/L and 500 mg/L); and (**D**) the NaCl resistance of the isolate L2 at different concentrations (2%, 4%, and 6.5%). The different letters on each bar represent significant difference between values (*p* < 0.05).

In order to ensure the better survival of the *L. paracasei* L2 strain during the gastro-intestinal transit and the fermentation process, the response to acid stress was analyzed. The effect of acidity on the viability of L2 was assessed. Figure 2A showed the survival rates of the L2 strain at various pH (2, 2.5,3 and 4) after 3 h of incubation. Results showed that the isolate achieved high survival rate up to 96.1% at pH4 after incubation for 2 h. The survival rate remained relatively high, above 76% after exposure to pH 3 for 2 h and 61% after 3 h. At pH2 and 2.5, there was no survival strain after 2 h and 3 h of incubation.

Bile tolerance

Screening new probiotic strains for bile salts tolerance was essential because it reflected the strains’ ability to survive in the intestine. Results from this test showed that *L. paracasei* L2 isolate was able to survive under 1% of ox gall concentration after 24 h and 48 h of incubation (Figure 2B). However, the growth rate decreased with the increase of the bile salts concentrations.

Lysozyme resistance

The lysozyme is a bacteriolytic enzyme commonly used in the food processing industry to control microbial growth. The resistance of the isolated *L. paracasei* L2 strain to various concentration lysozyme (100 mg/L, 200 mg/L, 300 mg/L and 500 mg/L) was determined in relation to the survival rate (Figure 2C). The tested isolate showed an important resistance to lysozyme even at a high concentration (500 mg/mL), reaching 100% after 24 h of incubation.

NaCl resistance

In order to analyze its response to saline stress, the NaCl resistance of the isolated strain was evaluated. Our results (Figure 2D) revealed that the growth of *L. paracasei* L2 decreased with the increase of the NaCl concentration (from 2% to 6.5%) in a dependent manner. The high growth rate (61.74%) was registered at 2% NaCl. However, it decreased significantly at 4%NaCl (*p* < 0.05).

### 3.3. Safety Tests

Antibiotics susceptibility test

The evaluation of the antibiotic resistance of probiotics should be measured for safety purposes. Our results showed that the *L. paracasei* L2 strain was found to be resistant to streptomycin, ciprofloxacin, and nalidixic acid (Table 1).

Hemolysis activity

*L. paracasei* showed a partial hemolysis (α-hemolysis). α-Hemolysis and γ-hemolysis were considered as safe organisms for human health because they were non-virulent, whereas β-hemolysis was considered harmful [6].

### 3.4. Antimicrobial Activity

To be used as a probiotic isolate, LAB microorganisms should have an antagonistic effect against microbial pathogens that are present in the host gastrointestinal tract. The results obtained from the agar well diffusion method revealed that the *L. paracasei* L2 showed a clear inhibition zone against the 8 pathogenic strains. Only the *Bacillus* was resistant as shown in Table 2. Based on these results, the *S. aureus* strain was found to be the most sensitive to L2 with a diameter of the clear inhibition zone above 19 mm.

### 3.5. DPPH Scavenging Activity

Antioxidant activity of LAB strains plays an important role in protection against free reactive oxygen species (ROS). The DPPH radical scavenging assay is widely used to evaluate the antioxidant activity as one of the evaluation standards for probiotic properties. In this context, the antioxidant activity was determined for the intact cells and the cell-free supernatants. Our results showed that the *L. paracasei* L2 intact cells (85.31%) had a higher antioxidant activity than the supernatant (51.72%) (Table 3).

### 3.6. Production of Enzymes

Enzyme production was an important criterion for the selection of probiotic strains to avoid the production of toxic substances [5,25]. Results obtained from the API ZYM kit (Table 4), demonstrated that *L. paracasei* L2 was capable of producing eight enzymes. The L2 strain did not produce ß–glucuronidase. This was a safety criterion since ß–glucuronidase is a bacterial carcinogenic enzyme that exerts negative effects on the liver [6].

### 3.7. Autoaggregation

**Figure 3:**
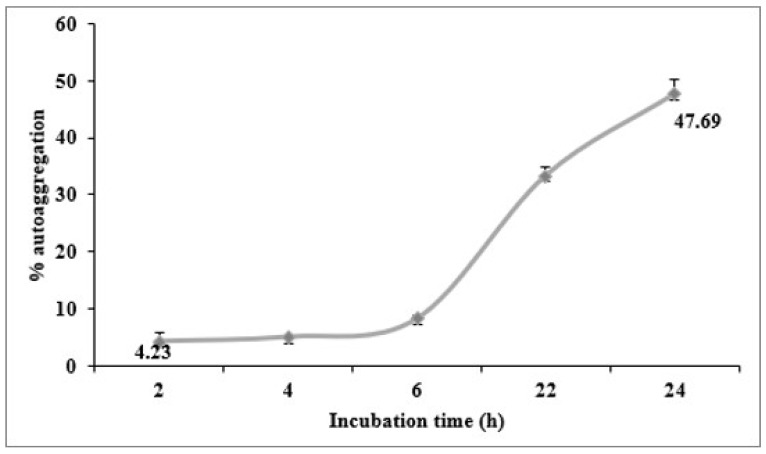
Aggregation ability of the selected *Lacticaseibacillus paracasei*. The values are represented as mean ± SD of three independent replicates Aggregation ability of the selected *L. paracasei* L2.

The auto-aggregation of probiotics was evaluated to investigate the colonization ability of the bacteria in the intestinal cells [26]. The results shown in Figure 3 revealed that the *L. paracasei* L2 aggregation percentage was found to be 4.25% after 4 h of incubation, which was the highest auto-aggregation reached 47.69% after 24 h incubation.

### 3.8. Hydrophobicity

**Figure 4:**
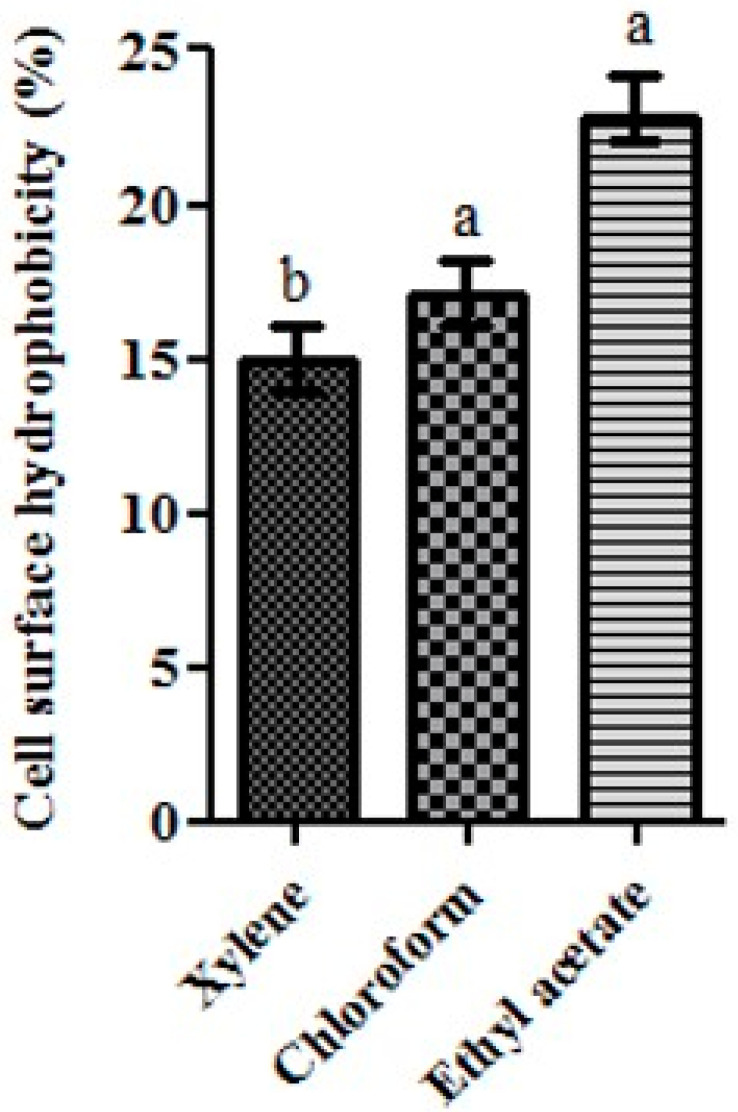
Cell surface hydrophobicity of *L. paracasei* L2 to solvents (Xylene, Chloroform, ethyl acetate). The different letters on each bar represent significant difference between values (*p* < 0.05).

The results of the cell surface hydrophobicity of the L2 strain showed that the highest percentage of hydrophobicity was 22.8% obtained with the ethyl acetate solvent (Figure 4); therefore, this strain, which was characterized as Lewis acid solvent solubility’s, appeared to be less hydrophobic [27].

### 3.9. Exopolysaccharides (EPS) Production

In recent years, EPS from LABs have attracted more attention because of their long history of safe use in substances aimed for human consumption such as the probiotics [28]. The L2 isolate strain from lben showed a ropy phenotype. The *L. paracasei* L2 produced viscous colonies on MRS agar plates that were supplemented with sucrose (2% *w/v*), indicating the possibility of exopolysaccharides production.

### 3.10. Biofilm Formation

The *lactobacillus* strains from the studied dairy products were strong producers of biofilm [29]. The biofilm formation potential of *L. paracasei* L2 isolated from Lben was evaluated both qualitatively and quantitatively. According to the color scale visualized on Conge red agar, the strain was found to be slime positive bacteria. Furthermore, the results from 1% Crystal violet (CV) staining assay showed that the isolate was found to form biofilm at the bottom of the well in the MRS broth under aerobic conditions. Based on the OD values, the *L. paracasei* L2 showed a high biofilm-formation ability (OD 570 > 1.7) after 24 h of incubation. Results are shown in Table 5.

### 3.11. In Vitro Adhesion Property to HCT-116 Cells

The adhesion ability of LAB strains is a prerequisite for the successful colonization of the human intestine epithelial cells and for the stimulation of the immune system. That is why, it’s important to evaluate it for its probiotic use [30]. Adherence to HCT-116 cells was examined by direct cell counting as shown in Table 5. Results showed that the strain had important adherence ability (45.03%). In our study, the adhesion ability of the *L. paracasei* L2 was stronger than the adhesion abilities of the other strains that had been investigated in previous studies [17].

### 3.12. Technological Characterization

Acidifying activity

The *L. paracasei* L2 had a low acidifying activity. After 24 h incubation, it had reached (ΔpH 0.53) and after 6 h (ΔpH 0.03). Results are presented in Table 5.

Autolytic activity

The autolysis activity of the isolated strain was (29.51%) (Table 5). According to other research findings, the *L. paracasei* L2 had a good autolytic property. The strains with autolysis rates between 25% and 65% had good autolytic properties [31].

## 4. Discussion

Currently, the most important and frequently used functional food compounds are probiotics. Probiotic bacteria have become continuously more popular over the past two decades due to the continued expansion of the scientific research into their beneficial effects on human health.

In consideration of this, and despite the strong scientific evidence associating to various health benefits of these microorganisms, the isolation and the characterization of new probiotic strains and evaluation of their safety and beneficial properties as a probiotic bacteria are important.

The results of the identification of L2 strain showed that this LAB strain isolated from lben belonged to *Lacticaseibacillus paracasei*, with 99% similarity to *L. paracasei*.

To be able to exert its beneficial effects as a new potential probiotic strain, it is expected to exhibit certain desirable characteristics. The ones currently determined by in vitro tests include: survival under GIT conditions such as acid tolerance, bile tolerance and lysozyme resistance.

In this setting, the most important characteristics of the probiotic LAB strains were their tolerance and survive in acidic conditions, and their ability to bear the initial acid stress [25]. In fact, the pH value in human stomach ranged from 1.5 during fasting to 4.5 after a meal, and food ingestion can take up to 3 h [32].

In the present study, the acid pH stability results of the tested *L. paracasei* L2 are consistent with previous research findings. In fact, it was reported that the *L. paracasei* strain that was isolated from ripened cheese and fermented beverage exhibited a good ability to grow after 24 h incubation at pH 3.5 [33]. In another study, Kumar et al. reported that the highest resistance of the LABs that were isolated from dairy samples was at pH 3 (60.52%). However, no isolate was found to be resistant to pH 2 [34]. The LAB isolates with a resistance to acid pH value 3 (≥50%) were considered acid tolerant [35]. More generally, the tolerance of the probiotic strains through the gastrointestinal tract depended not only on the incubation conditions but also on the tested strain [36].

For bile salts tolerance, it was reported that the resistance to bile salts varied considerably among the different species of *Lactobacillus* [30]. It also depended on the concentration as well as on the specific properties of the strain [32]. For instance, the *L. rhamnosus* strains from the traditional fermented mare milk exposed to low bile salts concentrations (0.3, 0.5 and 1%) reported stable maintenance of cell numbers [33]. Interestingly, the *L. paracasei* strains that were isolated from the dairy samples exhibited the highest level of bile salts tolerance with a resistance rate of ≥50% [33]. Therefore, *L. paracasei* L2 was expected to be able to reach the intestine. The findings of the present study suggest that the probiotic can adapt to the bile salts [5]. The concentration of bile salts in the intestine varied from 1.5% to 2% (*w/v*) during the first hour of digestion, and decreased afterwards to 0.3% (*w/v*) [32].

Furthermore, our findings revealed that *L. paracasei* had an important resistance to lysozyme which is known for its antibacterial activity against gram-positive bacteria. More specifically, this enzyme causes the degradation of peptidoglycan which represents the major part of the cell wall of this bacterial group [37].The resistance of this strain L2 was higher than that reported for *L. rhamnosus* strains in Riaz Rajoka et al.’s study [8]. The *L.hilgardii* strains that were isolated from Port wine were found to be highly resistant to lysozyme, surviving in concentrations as high as 1000 and 2000 mg/L [21].

Analyzing the response to saline stress of *L. paracasei* L2 is also important for the survival of the strain. It was reported that high amounts of NaCl might affect the cell viability of probiotics, especially in relation to the degree of survival and the activity of the LAB strains [38].

It was previously shown that the survival rate of LAB decreased with the increase of the sodium chloride levels greater than 3% (*w/w*) [38]. Nonetheless, it was demonstrated that the various *Lactobacillus* strains (*L. lactis*, *L. plantarum* and *L. brevis*) that were isolated from raw camel milk were able to grow at 4% of salt concentration [9]. Salt is largely used in the food industry as a taste enhancer or as a preservative.

For probiotic uses and safety purpose, the LAB strains must be resistant to certain antibiotics to allow them to survive in the gastrointestinal tract, especially when co-administrated with antibiotics [25]. In this context, the results of the antibiotics resistance analysis are consistent with those reported by Jan et al. [17]. However, Bengao et al. (2019) showed that *L. paracasei* was susceptible to streptomycin, kanamycin, and gentamycin. The LAB’s resistance to antibiotics was probably associated with their natural and intrinsic resistance due to membrane impermeability [34].

Moreover, *L. paracasei* L2 are considered as safe organisms for human health (α-hemolysis). The absence of a hemolytic activity was a prerequisite for safety as well as for the selection of new probiotic strains [39].

Furthermore, our results of antimicrobial activity were in accordance with other research findings that showed that the highest antibacterial effects of *L. salivarius* strains were on *S. aureus* [34,40]. The antimicrobial effect might be due to strain-specific properties or concentration-dependent conditions [40]. A wide range of antibacterial compounds were produced by *lactobacilli* strains, such as organic acids (lactic acid and acetic acid), bacteriocins, antifungal peptides/proteins, etc. [8,32,34].

The L2 strain did not produce ß–glucuronidase. This was a safety criterion since ß–glucuronidase is a bacterial carcinogenic enzyme that exerts negative effects on the liver [6].

Our strain L2 showed a high antioxidant activity for the intact cells than the supernatant of the culture. These results were in line with previous research findings showing that the highest radical scavenging capacity of *L. plantarum* MA2 was obtained by the intact cells when compared with supernatant culture and the cell free extract [41]. The antioxidant effects were reported to be strain-specific [17]. This effect was related to the cell surface active antioxidant enzymes, bioactive peptides, and exopolysaccharides which were present in the LAB cells [25,42]. However, the presence of enzymes, such as NADH-oxidase, SOD, NADH peroxide, and non-heme catalases played a major role in the LAB antioxidant activity [13].

To investigate the colonization ability of the bacteria in the intestinal cells [26]. The auto-aggregation ability of L2 was evaluated and the results in this present study was in accordance with previous reports showing that the highest auto-aggregation percentages of *L. fermentum* (IMAU60151) and *L. brevis* reached 51.5% and 52.5%, respectively, after 20 h to 24 h of incubation [17,32]. The auto-aggregation ability of the LAB cells is considered important in several ecological niches, especially in the human gut [32] and plays an important role in the GIT transit because it is related to the cell adherence properties [26].

Otherwise, the findings obtained of the cell surface hydrophobicity suggest that the chemical properties of the cell surface suggest that L2 might play protective and adhesion-promoting roles in the intestinal ecosystem [27]. Other research findings revealed that the highest hydrophobicity value of the LAB isolates (*L. plantarum*) was for n-hexadecane and xylene with values ranging from 19.4% to 46.2% [36]. Another LAB strain, *L. brevis*, showed higher affinity to chloroform than to ethyl acetate and xylene [17]. *L. lactis* displayed the lowest solubility in ethyl acetate as well as a low affinity to chloroform [5]. More generally, the cell surface hydrophobicity varied with the isolate and the tested solvent [26]. The hydrophobicity property was an important property for the probiotic bacteria in order to colonize the gastrointestinal tract (associated with its adhesion ability to cell surface especially intestinal epithelium) by exerting a beneficial effect, such as the exclusion of the enteropathogenic bacteria [26,36].

The possibility of exopolysaccharides production of L2 is in agreement with the research findings reported in Rajoka et al. study [8]. In fact, the exopolysaccharides (EPS) are biological extracellular sugar polymers with high-molecular weight that are produced during the metabolic process of microorganisms, such as bacteria [43]. EPS are important as they have potential health benefits for consumers (prebiotic potential, antimicrobial activity, and immunomodulatory activity). Moreover, they are used in the dairy fermentation industry for their texturizers and stabilizers characteristics as well as for their rheological properties [44].

The *lactobacillus* strains from the studied dairy products were strong producers of biofilm [29]. In contrast, the *L. paracasei* TD104 that was isolated from Chinese traditional fermented milk showed no ability to produce a biofilm after 24 h incubation [45]. However, another *L. paracasei* strain (LP10266) revealed a stronger biofilm formation ability [41]. Therefore, it can be concluded that the *L. paracasei* L2 that was isolated from Lben is able to grow as surface-attached biofilms. According to other studies, the bacterial biofilm production was strain dependent. For example, the *L. plantarum* J26 showed a more superior ability for biofilm formation than the *L. paracasei* TD104 that was tested under the same conditions [29,45]. Nevertheless, the biofilm formation can be affected by various factors, including the composition of the growth medium [46].

It is important to evaluate the adhesion ability of the strain for its probiotic use [30]. In our study, the adhesion ability of the *L. paracasei* L2 was stronger than the adhesion abilities of the other strains that had been investigated in previous studies [17].

In previous research, the level of the adhesion ability to cells varied from one study to another. This variation was observed in relation to the isolated LAB strains [17,20] and in relation to the matrix dependent [47]. It was also reported that the bacteria with a highly hydrophobic cell surface showed a higher adhesion ability to intestinal epithelium cells and helped to maintain bacterial cell adhesion [25]. Other researchers reported that the LAB strains were able to adhere to rat ileum epithelial cells, to chicken intestinal epithelium, and to human colorectal adenocarcinoma cells (Caco2) [20,30,34].

For a technological characterization, the acidifying activity and autolytic activity were evaluated.

The acidification rate varied among the different LAB strains. The strain with ΔpH of 0.4 units reached in 3, 3–5 or >5 h, were respectively considered as fast, medium, and slow. That is why *L. paracasei* L2 was characterized by a low acidifying activity. The slow acid-producing strains can be used as adjunct cultures, depending on their other important properties, such as proteolytic and autolytic activities [9]. It was shown that the LAB isolates had a high acidifying ability throughout the incubation period [36]. The acidifying activity of each strain was related to its specific ability to break down the carbon and nitrogen substrates in the medium as well as to its ability to assimilate the nutrients that were essential for growth [9].

The autolysis property is important especially for food industry. The cell lysis leads to the transformation of the bacterial intracellular enzymes into cheese for quick ripening and for the increase of the textural properties [23,48]. The autolysis of the *L. paracasei* L2 had good autolytic properties (the rate between 25% and 65%) [31].

There was a high degree of diversity of lysis among the *lactobacillus* strains. The highest one was recorded for *L. brevis* 90.77% [49]. The autolysis appeared to be a strain-dependent property [31].

## 5. Conclusions

For years, the lactobacillus strains have attracted researchers’ attention for their use as probiotics and for their applicability in the food industry. The isolate L2, isolated from lben, was identified as *Lacticaseibacillus paracasei* by the MALDI-TOF MS and by the 16S r-DNA sequencing. The L2 showed potential probiotic properties, such as acidity resistance, a very high resistance to lysozyme, and an important adherence to the epithelial intestinal cells. The isolate was resistant to three antibiotics out of the eight tested antibiotics. Additionally, the *L. paracasei* showed a non-hemolytic activity as well as an important antimicrobial activity against pathogens, especially against *Staphylococcus aureus*. Moreover, its antioxidant activity in DPPH radical scavenging was higher. Therefore, the *L. paracasei* L2 appears safe for use as a probiotic. It was essential to evaluate not only the probiotic characteristics for a new strain as a probiotic but also the technological properties, such as the acidifying activity, EPS production, and the autolytic activity. Results obtained from the present study showed that this strain can be proposed for use as a starter or as adjunct cultures for fermented foods.

## Figures and Tables

**Table 1:** Antibiotic resistance of the selected isolate indicated as ‘+’ resistance and ‘−’ susceptible and inhibitory zone (mm).

*L. paracasei* L2	Antibiotic (µg/mL)							
	NA (30 µg/mL)	S (10 µg/mL)	KAN (30 µg/mL)	CIP (5 µg/mL)	PE (10 µg/mL)	GEN (10 µg/mL)	TE (30 µg/mL)	E (15 µg/mL)
	0	0	13 ± 0.7	0	34 ± 0.4	15 ± 0.7	21 ± 0.7	24 ± 0.7
	+	+	−	+	−	−	−	−

±SD (n = 3). S: Streptomycin; TE: Tertarcyclin; PE: Penicillin; CIP: Ciprofloxacin; E: Erythromycin; GEN: Gentamycin; KAN: Kanamycin; NA: Nalidixic acid.

**Table 2:** Antimicrobial activity of the selected isolate against different pathogens (mm).

	*Lacticaseibacillus paracasei* L2
*Listeria monocytogenes* ATCC19115	10 ± 0.3
*Micrococcus luteus* NCIMB 8166	10 ± 0.3
*Enterococcus* *faecalis* ATCC 29212	12 ± 0.3
*Bacillus cereus* ATCC 11778	7 ± 0.5
*Staphylococcus aureus* ATCC 25923	19 ± 0.3
*Pseudomonas aeruginosa* ATCC 27853	11 ± 0.3
*Escherichia coli* ATCC 35218	11 ± 0.8
*Salmonella Typhi* ATCC 1408	10 ± 0.5

The antimicrobial activity is indicated as the diameter of the inhibiting zone (IZ). The values are expressed as mean ± SD (n =3).

**Table 3:** DPPH radical-scavenging activity.

Strain	DPPH Radical-Scavenging Activity (%)	
*L. paracasei* L2	Cell-free supernatant	Intact cells
	51.72 ± 0.5	85.31 ± 4.95

**Table 4:** Enzyme activity of *L. paracasei* measured using the API ZYM kit.

Enzymes	Substrat	Enzyme Activity	
Control	-	−	0
Alkaline phosphatase	2-naphthyl phosphate	−	0
Esterase	2-naphthyl butyrate	−	0
Esterase lipase	2-naphthyl caprylate	−	0
Lipase	2-naphthyl myristate	−	0
Leucine arylamidase	L-leucyl-2-naphthylamide	−	0
Valine arylamidase	L-valyl-2-naphthylamide	+	3
Cystine arylamidase	L-cystyl-2-naphthylamide	+	3
Trypsin	N-benzoyl-DL-arginine-2-naphthylamide	+	2
a-Chymotrypsin	N-glutaryl-phenylanine2-naphthylamide	+	1
Acid phosphatase	2-naphtyl phosphate	+	1
Naphthol-AS-BI-phosphohydrolase	Naphthol-AS-BIphosphate	+	1
α-Galactosidase	6-Br-2-naphthyl-aDgalactopyranoside	+	2
β-Galactosidase	2-naphthyl-bDgalactopyranoside	−	0
β-Glucuronidase	Naphthol-AS-BI-bDglucuronide	−	0
α-Glucosidase	2-naphthyl-aDglucopyranoside	−	0
β-Glucosidase	6-Br-2-naphthyl-bDglucopyranoside	−	0
N-Acetyl- b-glucosaminidase	1-naphthyl-N-acetyl-bDglucosaminide	+	4
α-Mannosidase	6-Br-2-naphthyl-aDmannopyranoside	−	0
α-Fucosidase	2-naphthyl-aLfucopyranoside	−	0

+: positive activity, −: negative activity. 0, 0 nmol; 1, 5 nmol; 2, 10 nmol; 3, 20 nmol; 4, 30 nmol.

**Table 5:** The acidifying, autolytic activities, biofilm formation and adhesion ability of the lactic acid bacteria isolated from Lben.

Strain	Acidifying Activity		Autolytic Activity	Biofilm Formation	Adhesion Ability to HCT-116 Cells
*L. paracasei* L2	ΔpH6	ΔpH24	29.51 ± 0.30	1.76 ± 0.45	45.03 ± 2.77
	0.03b ± 0.06	0.53a ± 0.06			
